# Repetitive Transcranial Magnetic Stimulation for Improving Cognitive Function in Patients With Mild Cognitive Impairment: A Systematic Review

**DOI:** 10.3389/fnagi.2020.593000

**Published:** 2021-01-14

**Authors:** Lijuan Jiang, Huiru Cui, Caidi Zhang, Xinyi Cao, Nannan Gu, Yikang Zhu, Jijun Wang, Zhi Yang, Chunbo Li

**Affiliations:** ^1^Shanghai Key Laboratory of Psychotic Disorders, Shanghai Mental Health Center, Shanghai Jiao Tong University School of Medicine, Shanghai, China; ^2^Institute of Psychology and Behavioral Science, Shanghai Jiao Tong University, Shanghai, China; ^3^Center for Excellence in Brain Science and Intelligence Technology (CEBSIT), Chinese Academy of Science, Beijing, China; ^4^Brain Science and Technology Research Center, Shanghai Jiao Tong University, Shanghai, China; ^5^Laboratory of Psychological Heath and Imaging, Shanghai Mental Health Center, Shanghai Jiao Tong University School of Medicine, Shanghai, China

**Keywords:** repetitive transcranial magnetic stimulation, mild cognitive impairment, cognitive function, systematic review, meta-analysis

## Abstract

**Background:** Mild cognitive impairment (MCI) is an early stage of Alzheimer's disease. Repetitive transcranial magnetic stimulation (rTMS) has been widely employed in MCI research. However, there is no reliable systematic evidence regarding the effects of rTMS on MCI. The aim of this review was to evaluate the efficacy and safety of rTMS in the treatment of MCI.

**Methods:** A comprehensive literature search of nine electronic databases was performed to identify articles published in English or Chinese before June 20, 2019. The identified articles were screened, data were extracted, and the methodological quality of the included trials was assessed. The meta-analysis was performed using the RevMan 5.3 software. We used the GRADE approach to rate the quality of the evidence.

**Results:** Nine studies comprising 369 patients were included. The meta-analysis showed that rTMS may significantly improve global cognitive function (standardized mean difference [SMD] 2.09, 95% confidence interval [CI] 0.94 to 3.24, *p* = 0.0004, seven studies, *n* = 296; low-quality evidence) and memory (SMD 0.44, 95% CI 0.16 to 0.72, *p* = 0.002, six studies, *n* = 204; moderate-quality evidence). However, there was no significant improvement in executive function and attention (*p* > 0.05). Subgroup analyses revealed the following: (1) rTMS targeting the left hemisphere significantly enhanced global cognitive function, while rTMS targeting the bilateral hemispheres significantly enhanced global cognitive function and memory; (2) high-frequency rTMS significantly enhanced global cognitive function and memory; and (3) a high number of treatments ≥20 times could improve global cognitive function and memory. There was no significant difference in dropout rate (*p* > 0.05) between the rTMS and control groups. However, patients who received rTMS had a higher rate of mild adverse effects (risk ratio 2.03, 95% CI 1.16 to 3.52, *p* = 0.01, seven studies, *n* = 317; moderate-quality evidence).

**Conclusions:** rTMS appears to improve global cognitive function and memory in patients with MCI and may have good acceptability and mild adverse effects. Nevertheless, these results should be interpreted cautiously due to the relatively small number of trials, particularly for low-frequency rTMS.

## Introduction

Mild cognitive impairment (MCI) is the transitional stage between normal aging and dementia, in which individuals have subjective cognitive deficits and objective memory impairment without impairments in daily activities (Petersen et al., 1999). Along with cognitive decline, MCI implies an increased risk of falls, slow walking speed, and physical frailty (LeWitt et al., 2020; Ma and Chan, 2020). The mechanisms underlying cognitive decline are multifactorial, including inflammation, impaired hypothalamic-pituitary axis stress response, imbalanced energy metabolism, mitochondrial dysfunction, oxidative stress, and endocrine dysfunction (Zou et al., 2018; Xu et al., 2019a; Li et al., 2020; Ma and Chan, 2020; Wang et al., 2020a). The annual conversion rate of MCI to dementia ranges from 10 to 15%, demonstrating that it is an important condition to identify and treat (Petersen et al., 2009; Ding et al., 2016). Existing pharmacological interventions for MCI are unsatisfactory and have limited effectiveness (Sanford, 2017). Therefore, non-pharmacological interventions for MCI have received increasing attention (Feng et al., 2018; Kasper et al., 2020).

Recently, the use of non-invasive brain stimulation has garnered considerable clinical and research interest (Hsu et al., 2015; Xu et al., 2019b). Repetitive transcranial magnetic stimulation (rTMS) is a non-invasive method of brain stimulation in which a train of magnetic pulses is delivered to a specific target location of the brain (Wei et al., 2017). rTMS involves trains of magnetic pulses of various frequencies and intensities. As a general rule, high frequencies (≥5 Hz) increase cortical excitability and low frequencies (≤1 Hz) suppress it (Lin et al., 2019). rTMS has been widely studied in patients with various neuropsychiatric illnesses such as depression, epilepsy, Parkinson's disease, and schizophrenia (Najib et al., 2011; Guo et al., 2017).

In recent years, several meta-analyses have investigated the effects of rTMS in older patients with cognitive impairment, demonstrating that rTMS may have a beneficial effect on cognitive function (Hsu et al., 2015; Wang et al., 2015, 2020b; Cheng et al., 2018; Dong et al., 2018; Lin et al., 2019; Chou et al., 2020). Four reviews focused on patients with Alzheimer's disease (AD) (Hsu et al., 2015; Dong et al., 2018; Lin et al., 2019; Wang et al., 2020b), and two reviews included MCI and AD patients, but did not analyze MCI patients separately (Cheng et al., 2018; Chou et al., 2020). Moreover, only one review in China comprising small sample sizes has investigated the effects of rTMS in patients with MCI (Wang et al., 2015). Despite the growing body of evidence supporting the beneficial effects of rTMS in older patients with cognitive impairment, the relationships between the effect of rTMS and factors such as target site, parameter settings, and treatment course still require further investigation. Therefore, the aim of this systematic review and meta-analysis was to provide up-to-date evidence on the effects of rTMS on cognitive function in MCI patients.

## Materials and Methods

This work adhered to the Preferred Reporting Items for Systematic Reviews and Meta-Analyses (PRISMA) guidelines (Liberati et al., 2009) and was registered in the open access database International Prospective Register of Systematic Reviews (PROSPERO) (http://www.crd.york.ac.uk/PROSPERO/; registration number: CRD42019126269).

### Search Strategies

The following databases were searched to identify studies on the effect of rTMS on MCI, published before June 20, 2019: PubMed, ISI Web of Science, Embase, the Cochrane Library, EBSCO, Chinese National Knowledge Infrastructure (CNKI), Chinese Technical Periodicals (VIP), Wanfang Database, and China BioMedical Literature database (SinoMed). The English keywords used for the database searches were “mild cognitive impairment,” “MCI,” “transcranial magnetic stimulation,” “repetitive transcranial magnetic stimulation,” “TMS,” and “rTMS.” The Chinese keywords were “Qingdurenzhizhangai,” “Qingdurenzhisunhai,” “Chongfujingluciciji.” The reference lists of identified articles were checked for other potential studies.

### Inclusion and Exclusion Criteria

To be included, studies had to meet the following criteria: (1) they were randomized controlled studies investigating the effects of rTMS treatment on the cognitive function of patients with MCI; (2) they included participants diagnosed with MCI based on any diagnostic criteria, such as the Petersen criteria, the National Institute on Aging-Alzheimer's Association criteria for MCI due to AD (Albert et al., 2011), or the fifth edition of the Diagnostic and Statistical Manual of Mental Disorders; (3) the experimental group received rTMS, regardless of stimulation site and stimulation frequency. rTMS could be combined with other interventions such as drug therapy or cognitive training; (4) the control group received sham rTMS stimulation, medication, or other interventions; (5) only the first treatment period of cross-over trials was considered; (6) outcomes included global cognitive ability and specific domain of cognition, which were measured by neuropsychological tests or other objective measurements.

Studies were excluded if (a) they were animal studies; (b) included participants with vascular cognitive impairment or other neurological disorders resulting from dementia or Parkinson's disease; (c) they were reviews, conference presentations, or unpublished reports; (d) they were duplicate reports; (e) they used a blank control as their control group; or (f) they just had a single TMS pulse intervention or studies with ≤ 1 week of intervention time.

### Evaluation of the Quality of Studies

We evaluated the quality of included studies based on the criteria specified in the Cochrane Handbook for Systematic Reviews of Interventions (Higgins and Green, 2011) and the Grading of Recommendations, Assessment, Development, and Evaluation (GRADE) framework (Guyatt et al., 2008). Two researchers (LJ and CZ) independently extracted data from each included study and compared their results. When discrepancies occurred, they discussed their differences and a consensus was reached; if necessary, a third researcher was asked to resolve any remaining differences. If possible, the authors of the original article were contacted if the information was unclear or insufficient.

### Evaluation of Risk of Bias

The risk of bias was assessed using the method recommended by the Cochrane Collaboration (Higgins and Green, 2011). The following characteristics were evaluated: (a) adequacy of sequence generation; (b) allocation concealment; (c) use of blinding; (d) how incomplete outcome data (dropouts) were addressed; (e) evidence of selective outcome reporting; and (f) other potential risks that may harm the validity of the study. The risk of bias for each domain was graded as low, high, or unclear.

### Evaluation of Quality of Evidence

The GRADE approach (Guyatt et al., 2008) was used to categorize the quality of evidence provided in each report into four levels: (a) “high quality”: further research was unlikely to affect the reliability of the efficacy evaluation results; (b) “medium quality”: further research was likely to affect the reliability of the efficacy evaluation results and very likely to change the outcome of the evaluation; (c) “low quality”: further research was very likely to affect the reliability of the efficacy evaluation results and the evaluation outcome was very likely to change; and (d) “very low quality”: results of any efficacy evaluation were uncertain. GRADEpro software (McMaster University, Hamilton, Canada) was used to edit, analyze, and graph the level of evidence. If a randomized controlled trial was defective, the quality of the evidence was downgraded by one or two levels.

### Data Extraction

A data extraction table was constructed and two researchers extracted and double-checked data from the included articles. The following information was extracted: basic study information (study authors, year of publication, study design), participant characteristics (age, and sample size), rTMS parameters [stimulus site, true stimulus frequency, stimulus intensity (% of resting motor threshold), and treatment regimen], cognitive outcome measures, dropout rate, and adverse effects. Motor threshold (MT) was defined as the minimum stimulator intensity capable of inducing a visible muscle twitch of the contralateral hand in at least 50% of a series of ten single-pulse TMS trials (Rossini et al., 1994). Cognitive outcomes were classified into three cognitive domains: (1) global cognitive function [e.g., the Mini-Mental State Examination (Folstein et al., 1975)]; (2) memory (encoded, stored, and retrieved of information); (3) executive function and attention (multi-dimensional and complex cognitive constructs) (Karssemeijer et al., 2017). If cognitive outcome data were reported from multiple time points, those from immediately after the intervention were obtained for meta-analysis.

### Data Analysis

RevMan 5.3 statistical software (The Nordic Cochrane Center, The Cochrane Collaboration, Copenhagen, Denmark) was used to conduct statistical analyses. Quantitative variables were summarized using standardized mean differences (SMDs); qualitative variables were summarized using risk ratios (RRs). Corresponding 95% confidence intervals (CIs) were also calculated. Pooled results were presented using forest plots. The degree of heterogeneity was evaluated using the Q statistic generated from the χ^2^ test. The degree of statistical heterogeneity was assessed by the *I*^2^ statistic. Studies with *I*^2^ < 50% and *p* ≥ 0.1 were considered homogeneous and a fixed-effects model was used; studies with *I*^2^ > 50% or *p* < 0.1 were considered heterogeneous and a random-effects model was used. Subgroup analyses were performed separately according to stimulus site, stimulus frequency, and treatment course. We also performed sensitivity analysis if necessary. Funnel plots were used to assess the possibility of publication bias.

## Results

### Search and Selection of Studies

**Figure 1:**
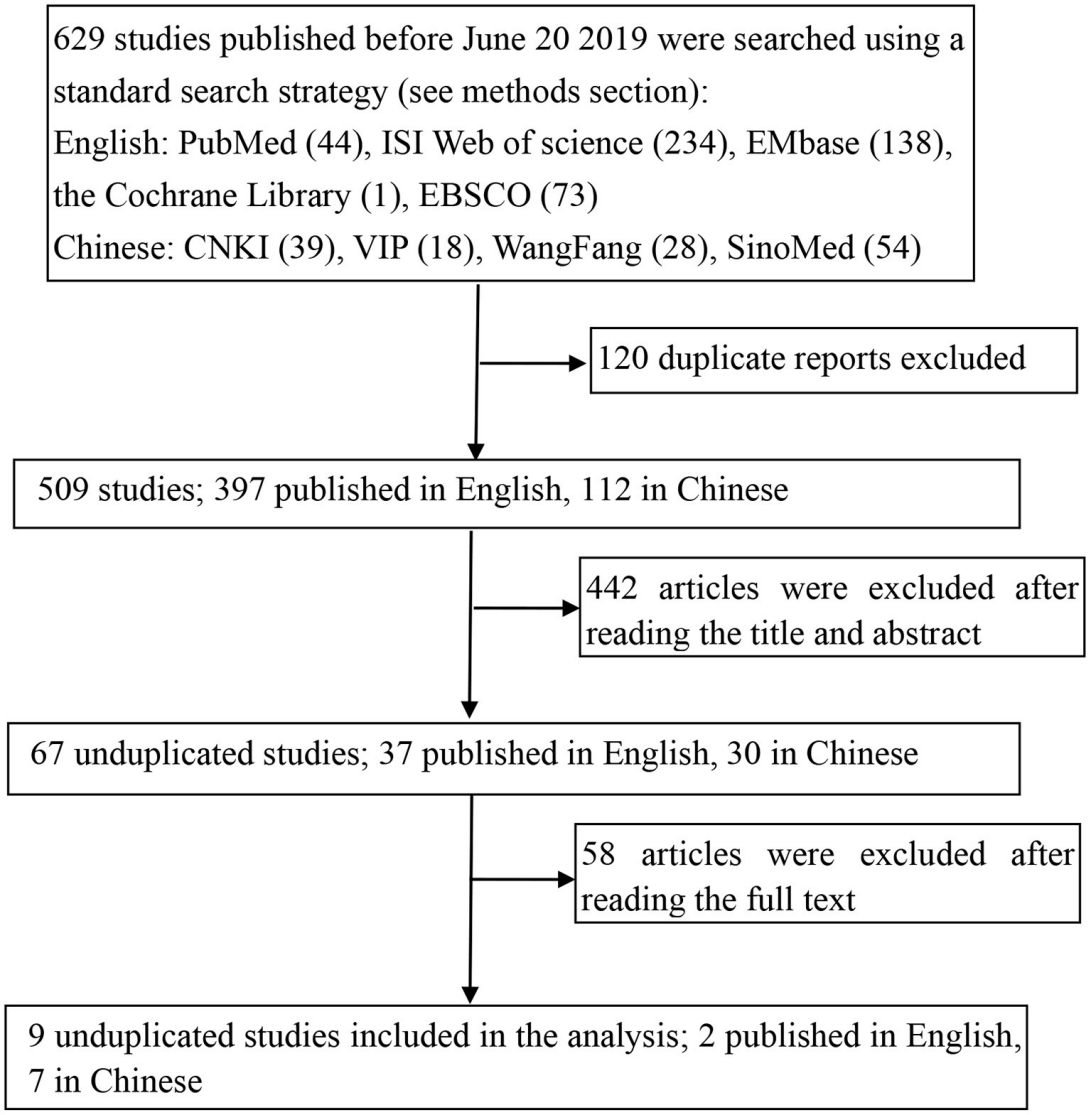
Flowchart of the literature search and screening processes.

The study selection process is shown in Figure 1. A total of 629 potentially relevant studies were identified from five English and four Chinese databases using relevant search strategies. Of these relevant studies, 120 duplicates were removed, and the eligibility of the remaining 509 studies was further assessed. During the title and abstract screening phase, an additional 442 articles were removed. Finally, after the full texts of the remaining 67 articles were reviewed, 58 articles were excluded, thus nine studies were included in this meta-analysis.

### Characteristics of the Included Studies

Table 1 shows the characteristics of the nine studies included in this meta-analysis, comprising a total of 369 participants (187 in the rTMS group and 182 in the control group). Two trials (Drumond Marra et al., 2015; Cui et al., 2019) were in English, and the remaining were in Chinese. Participants were diagnosed with MCI based on one of the following diagnostic criteria: the Petersen criteria, European Consortium on Alzheimer's Disease criteria, American Society of Neuroscience Quality Standards Branch criteria, National Institute on Aging-Alzheimer's Association criteria, or Chinese expert consensus on the prevention and treatment of cognitive dysfunction criteria. Only one study included low-frequency rTMS (Wan et al., 2016); all other studies used high-frequency rTMS (Han et al., 2013; Yang et al., 2014; Zhang et al., 2014; Drumond Marra et al., 2015; Sun and Ma, 2015; Long et al., 2016; Wen et al., 2018; Cui et al., 2019). rTMS stimulation sites included the left dorsolateral prefrontal cortex (DLPFC) (Drumond Marra et al., 2015; Sun and Ma, 2015; Long et al., 2016; Wen et al., 2018), right DLPFC (Cui et al., 2019), bilateral DLPFC (Han et al., 2013; Yang et al., 2014), bilateral prefrontal cortex (PFC) (Zhang et al., 2014), and bilateral anterior temporal lobe (Wan et al., 2016), with a stimulation intensity from 80 to 110% resting MT. The repetition number of intervention was 10–48 times. The interventions in the control group, which included sham rTMS stimulation [three studies used sham coil (Yang et al., 2014; Drumond Marra et al., 2015; Long et al., 2016); three studies rotated the coil 90 degrees to achieve the effect of sham therapy (Han et al., 2013; Wen et al., 2018; Cui et al., 2019)], medication treatment (Zhang et al., 2014; Wan et al., 2016), and cognitive training (Sun and Ma, 2015).

**Table 1:** Characteristics of the nine included studies.

**References**	**Study design**	**Interventions**	**Age (M ± SD)**	**Sample size (M/F)**	**Site for stimulation**	**Stimulation frequencyStimulation intensity (%MT)**	**Treatment frequency, Number of pulses each time**	**Cognitive outcomes/Measure**
Han et al., 2013	Parallel	T: active rTMS C: sham rTMS	66.5 (5.02) 66.7 (5.25)	22 (8/14) 18 (6/12)	Bilateral DLPFC	20 Hz 80%	40 times 36,000 pulses	Global cognitive function: MoCA; Memory: associative learning test, episodic memory test; Executive function and attention: TMT-A, WCST, VFT, DSST
Yang et al., 2014	Parallel	T: active rTMS C: sham rTMS	66 (6) 66 (7)	18 (8/10) 15 (7/8)	Bilateral DLPFC	20 Hz 80%	40 times 36,000 pulses	Global cognitive function: MMSE
Zhang et al., 2014	Parallel	T: active rTMS C: piracetam treatment	65.6 (8.9) 65.9 (9.9)	25 (12/13) 25 (13/12)	Bilateral PFC	5 Hz 100%	24 times 800 pulses	Global cognitive function: MoCA
Drumond Marra et al., 2015	Parallel	T: active rTMS C: sham rTMS	65.1 (3.5) 65.2 (4.1)	15 (6/9) 19 (6/13)	Left DLPFC	10 Hz 110%	10 times 2,000 pulses	Memory: RBMT, WMS, WAIS-III; Executive function and attention: TMT-B, VFT
Sun and Ma, 2015	Parallel	T: active rTMS + cognitive training C: cognitive training	65.4 (5.6) 63.4 (8.2)	40 (23/17) 40 (20/20)	Left DLPFC and left PFC	15 Hz 80–110%	48 times NA	Global cognitive function: MoCA
Long et al., 2016	Parallel	T: active rTMS C: sham rTMS	68.27 (9.85) 65.63 (9.36)	15 (8/7) 15 (6/9)	Left DLPFC	15 Hz 90%	10 times 1,000 pulses	Global cognitive function: MoCA; Memory: CMS
Wan et al., 2016	Parallel	T: active rTMS + conventional drug treatment C: conventional drug treatment	65.44 (9.61) 65.11 (5.39)	18 (12/6) 18 (11/7)	Bilateral Anterior temporal	1 Hz 80%	15 times 600 pulses	Memory: CMS
Wen et al., 2018	Parallel	T: active rTMS C: sham rTMS	64.17 (5.21) 65.91 (4.93)	23 (14/9) 22 (10/12)	Left DLPFC	10 Hz 80%	20 times 400 pulses	Global cognitive function: MoCA; Memory: RBMT
Cui et al., 2019	Parallel	T: active rTMS C: sham rTMS	73.91 (10.01) 74.00 (7.62)	11 (3/8) 10 (5/5)	Right DLPFC	10 Hz 90%	10 times 1,500 pulses	Global cognitive function: MMSE, ACE-III; Memory: logic memory test, AVLT; Executive function and attention: TMT-A, TMT-B

*ACE-III, Addenbrooke's Cognitive Examination-III; AVLT, auditory verbal learning test; CMS, Clinical Memory Scale; DLPFC, Dorsolateral Prefrontal Cortex; DSST, Digit Symbol Substitution Test; F, female; M, male; M, mean; MT, Motor Threshold; MMSE, Mini-Mental State Examination; MoCA, Montreal Cognitive Assessment; NA, no data or not describe; RBMT, Rivermead Behavioral Memory Test; rTMS, repetitive Transcranial Magnetic Stimulation; SD, Standard deviation; TMT, Trial Making Tests; VFT, Verbal Fluency Test; WAIS-III, Wechsler Adult Intelligence Scale III; WCST: Wisconsin Card Sorting Test; WMS, Wechsler Memory Scale*.

With respect to outcome measures, different cognitive measurement tools were applied to assess the same cognitive domains within a study or among studies. Measures of global cognitive function included the Montreal Cognitive Assessment (MoCA) (five studies) (Han et al., 2013; Zhang et al., 2014; Sun and Ma, 2015; Long et al., 2016; Wen et al., 2018), and Mini-Mental State Examination (MMSE) (two studies) (Yang et al., 2014; Cui et al., 2019). Memory was measured using the associative learning test (one study) (Han et al., 2013), Rivermead Behavioral Memory Test (RBMT) (two studies) (Drumond Marra et al., 2015; Wen et al., 2018), Clinical Memory Scale (CMS) (two studies) (Long et al., 2016; Wan et al., 2016), logic memory test (one study) (Cui et al., 2019). To assess executive function and attention two studies used the Trial Making Test-A (TMT-A) (Han et al., 2013; Cui et al., 2019), two studies used the Trial Making Test-B (TMT-B) (Drumond Marra et al., 2015; Cui et al., 2019), and two studies used the verbal fluency test (VFT) (Han et al., 2013; Drumond Marra et al., 2015) (Table 2).

**Table 2:** GRADE quality of evidence assessment of individual outcome indicators for the efficacy of repetitive transcranial magnetic stimulation in the treatment of mild cognitive impairment.

**Outcome indicator**	**Number of included cases**	**Heterogeneity**		**Model of analysis**	**Group effect value**		**Estimated value**	**95% CI**	**Grade**
		***I^**2**^***	***p***		***Z***	***p***			
Global cognitive function	296	93%	<0.00001	Random effect	3.55	0.0004	2.09 (SMD)	0.94, 3.24	Low
Memory	204	26%	0.24	Fixed effect	3.07	0.002	0.44 (SMD)	0.16, 0.72	Moderate
Executive function and attention	93	49%	0.14	Fixed effect	0.92	0.36	−0.19 (SMD)	−0.61, 0.22	Moderate
Drop-out rate	260	7%	0.37	Fixed effect	0.01	0.99	0.99 (RR)	0.39, 2.54	Moderate
Adverse effect	317	0%	0.66	Fixed effect	2.50	0.01	2.03 (RR)	1.16, 3.52	Moderate

*SMD, standardized mean difference; RR, relative risk; CI, confidence interval; GRADE, Grading of Recommendation Assessment, Development and Evaluation*.

### Research Quality

**Figure 2:**
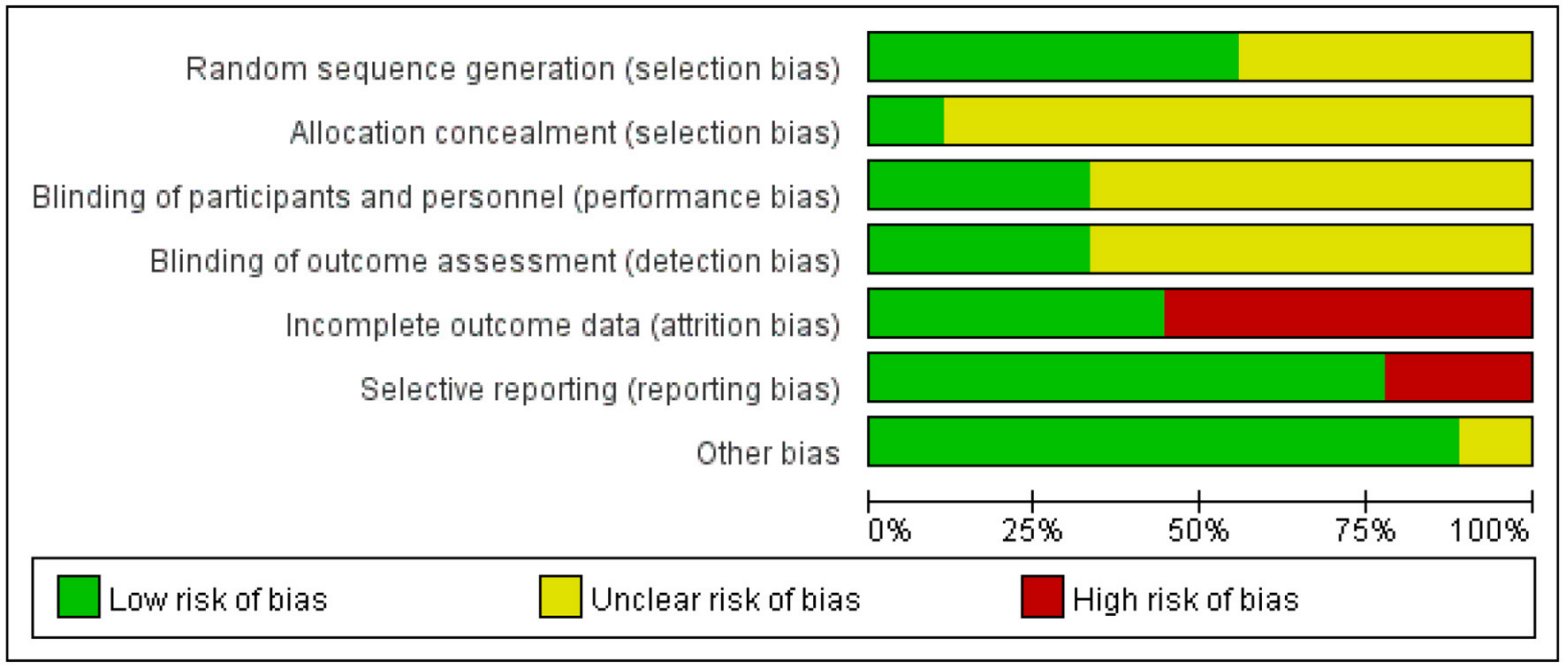
Risk of bias graph: review authors' judgements about each risk of bias item presented as percentages across all included studies.

The summary of the risk of bias of the included studies is shown in Figure 2. All included trials reported random allocation, but only five of the nine studies described the method used to generate the random sequence in detail and were thus rated “low risk” (Yang et al., 2014; Drumond Marra et al., 2015; Wan et al., 2016; Wen et al., 2018; Cui et al., 2019). Only one study described the allocation concealment procedure in detail (Drumond Marra et al., 2015). In three studies, the participants and researchers were blinded, thus performance bias was rated as “low risk” (Yang et al., 2014; Drumond Marra et al., 2015; Cui et al., 2019). The risk of attrition bias in five studies was rated as “high risk” because the research data were incomplete (due to drop-out and adverse effects) and intention-to-treat analysis was not implemented (Han et al., 2013; Yang et al., 2014; Drumond Marra et al., 2015; Sun and Ma, 2015; Cui et al., 2019). Two studies were selective in reporting their results, thus reporting bias was rated as “high risk” (Drumond Marra et al., 2015; Cui et al., 2019). Studies with unclear information were rated as “unclear risk.”

### Meta-Analysis of Treatment Effect

#### Global Cognitive Function

**Figure 3:**
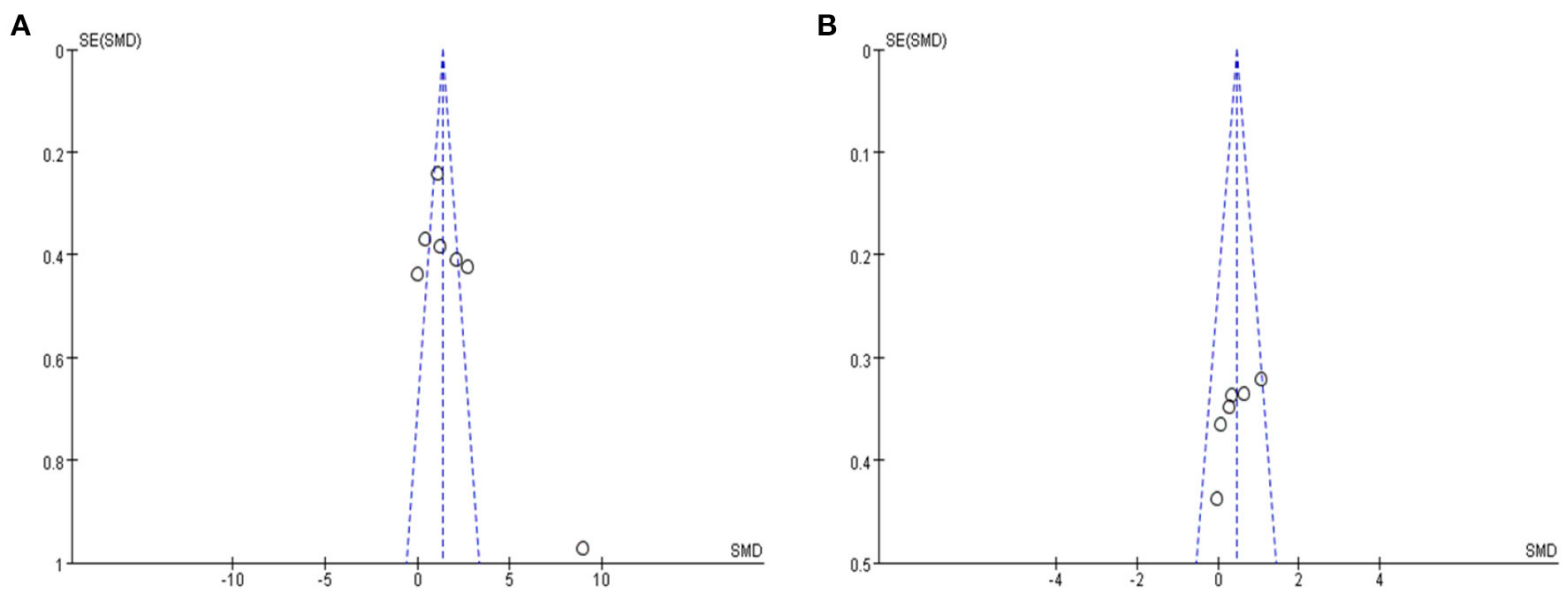
**(A)** Funnel plot for the publication bias of global cognitive function **(B)** Funnel plot for the publication bias of memory. SMD, standardized mean difference.

**Figure 4:**
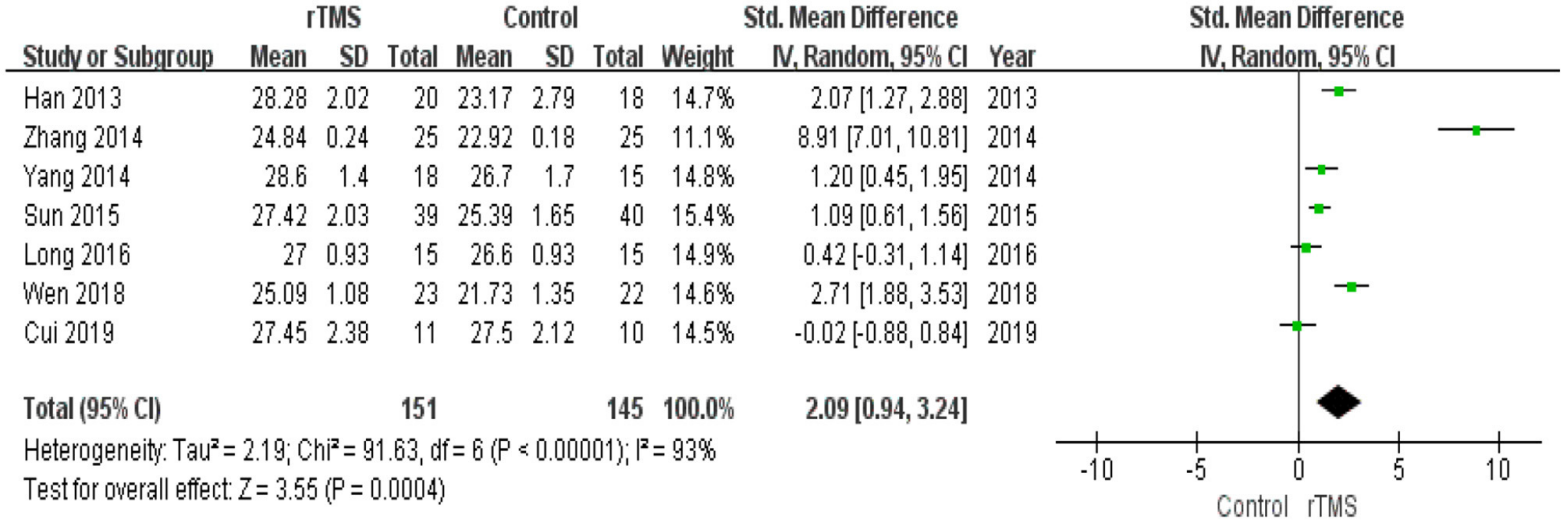
Forest plot of the comparison between the repetitive transcranial magnetic stimulation and control groups with respect to global cognitive function.

Seven studies with a total of 296 participants with MCI assessed the effects of rTMS on global cognitive ability. The heterogeneity of the included studies was high (*I*^2^ = 93%, *p* < 0.00001), so a random-effects model was used for the meta-analysis. The funnel plot revealed significant asymmetry (Figure 3A). However, it is usually recommended that ≥ 10 studies are required to make a definitive conclusion about publication bias, so this result may be considered suggestive of, but not definite evidence of, publication bias. Due to the high heterogeneity between studies, a sensitivity analysis was conducted. Omitting one study with low standard deviation (Zhang et al., 2014) did not significantly alter the pooled SMD. The combined results demonstrated that the rTMS group had significantly improved global cognitive function (SMD 2.09, 95% CI 0.94 to 3.24, *p* = 0.0004) compared to the control group (Figure 4). According to the GRADE system, the overall level of evidence with respect to the effect of rTMS on global cognitive function was “low” (Table 2).

#### Subgroup Analysis of Global Cognitive Function

**Figure 5:**
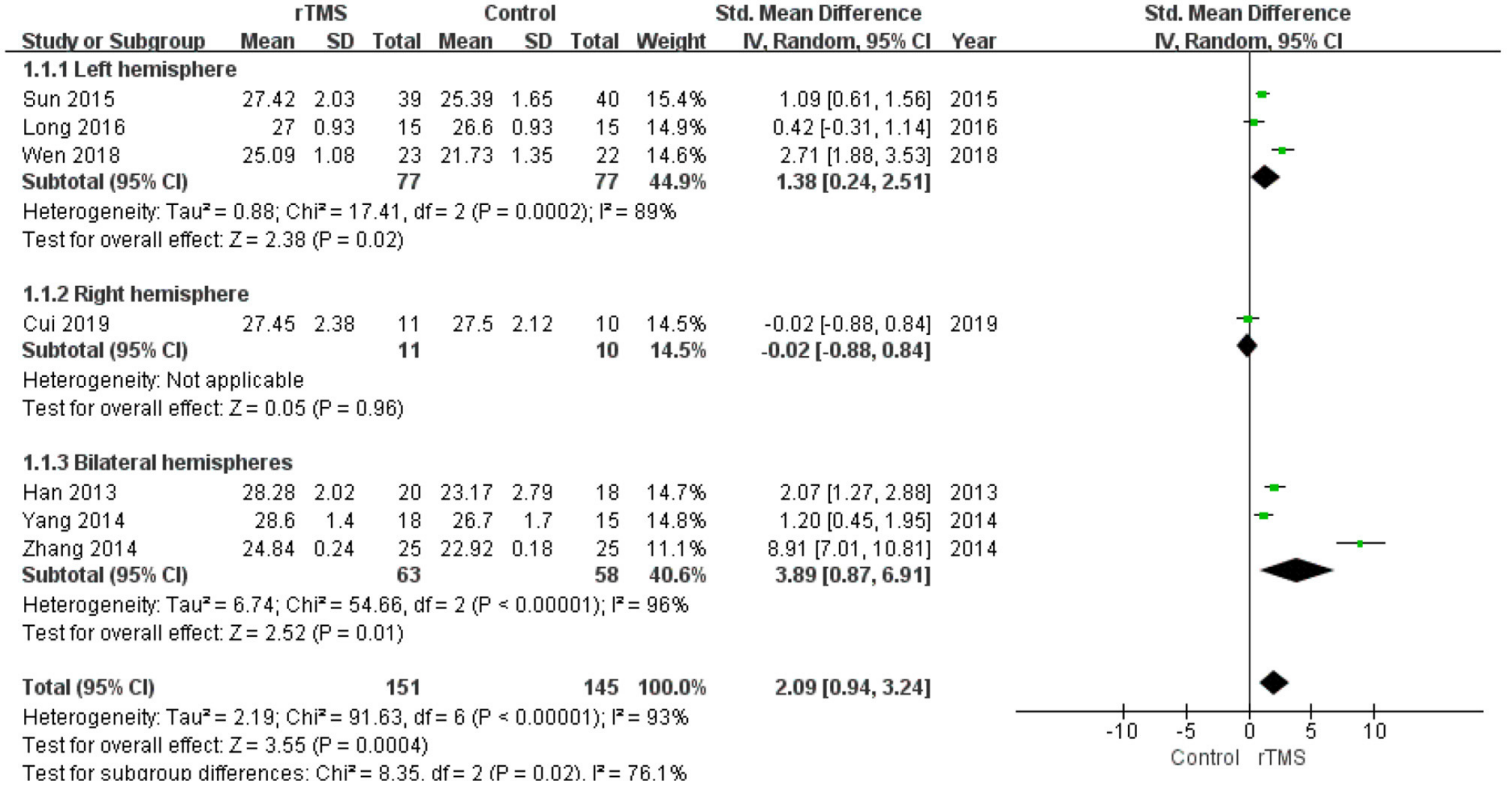
Meta-analysis forest plot of subgroup analysis showing global cognitive function of repetitive transcranial magnetic stimulation group vs. control group in the treatment of mild cognitive impairment: left hemisphere vs. right hemisphere vs. bilateral hemispheres.

A subgroup analysis was conducted according to the stimulation site (the left hemisphere, right hemisphere, and bilateral hemispheres). The subgroup analysis revealed a standardized mean difference of 1.38 (95% CI 0.24 to 2.51) for trials involving “left hemisphere” stimulation and a standardized mean difference of −0.02 (95% CI −0.88 to 0.84) for trials involving “right hemisphere” stimulation. The mean effect size for trials involving “bilateral hemispheres” stimulation was 3.89 (95% CI 0.87 to 6.91) (Figure 5).

**Figure 6:**
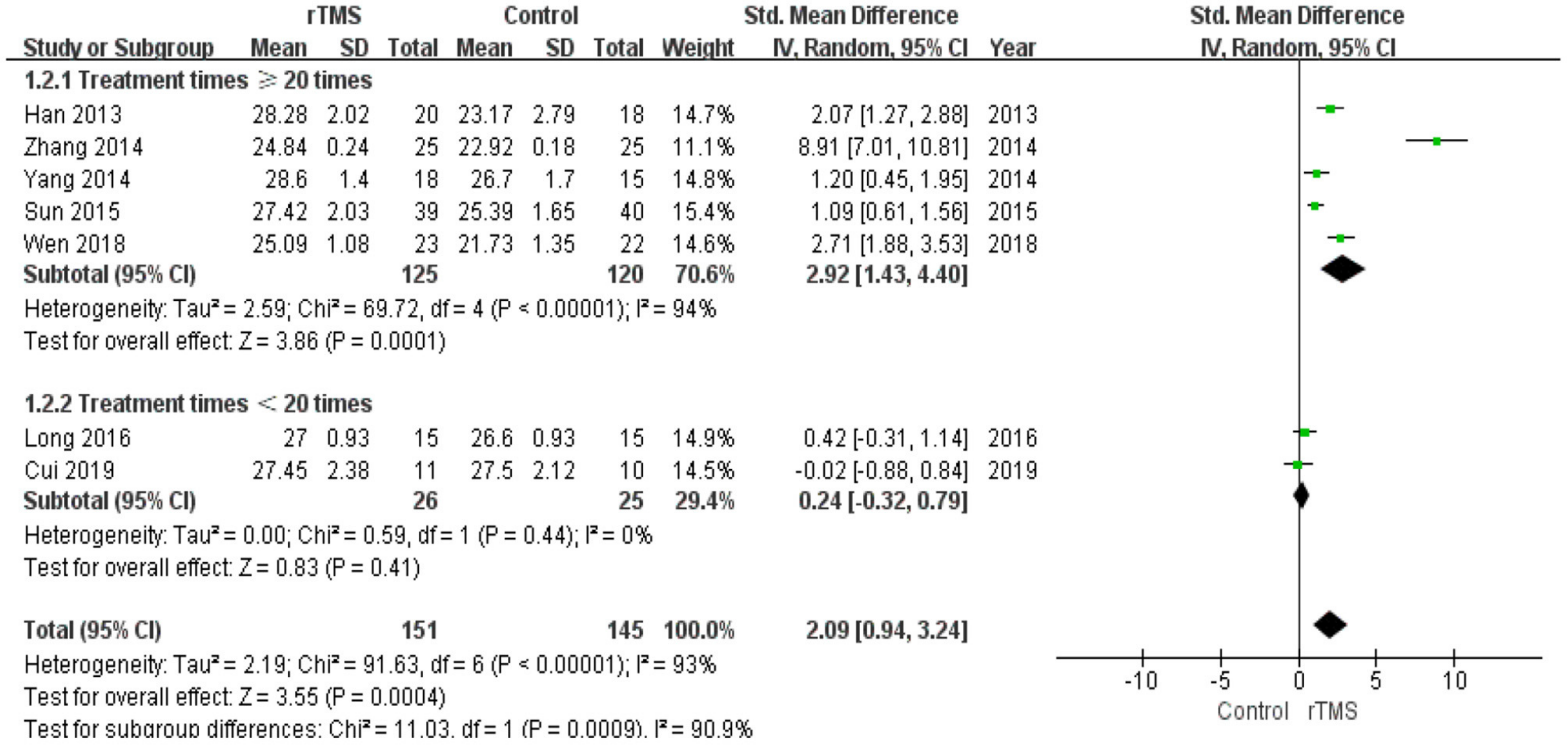
Meta-analysis forest plot of subgroup analysis showing global cognitive function of repetitive transcranial magnetic stimulation group vs. control group in the treatment of mild cognitive impairment: treatment times ≥20 times vs. treatment times < 20 times.

The number of treatment sessions in the included studies ranged from 10 to 48 times. We divided the studies into two groups: those with a high number of treatments (≥20 times) and those with a low number of treatments (< 20 times). The subgroup analysis revealed that the standardized mean difference for studies with a high number of treatments was 2.92 (95% CI 1.43 to 4.40). Studies with a low number of treatments showed a standardized mean difference of 0.24 (95% CI −0.32 to 0.79). These results indicate that a high number of rTMS treatments produced better global cognitive function than a low number of rTMS treatments (Figure 6).

#### Memory

**Figure 7:**
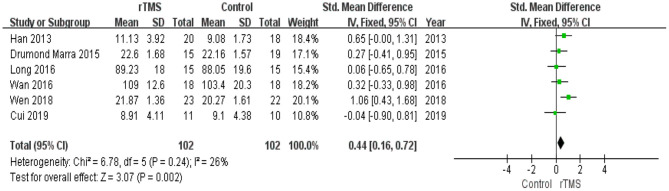
Forest plot of the comparison between the repetitive transcranial magnetic stimulation and control groups with respect to memory.

Six studies reported the effects of rTMS on memory. The heterogeneity of the included studies was low (*I*^2^ = 26%, *p* = 0.24); therefore, a fixed-effects model was applied. The funnel plot did not reveal significant asymmetry (Figure 3B). The results of the meta-analysis showed that rTMS had a significant effect on memory improvement (SMD 0.44, 95% CI 0.16 to 0.72, *p* = 0.002) compared to the control group (Figure 7). According to the GRADE system, the overall level of evidence with respect to the effect of rTMS on memory was “moderate” (Table 2).

#### Subgroup Analysis of Memory

**Figure 8:**
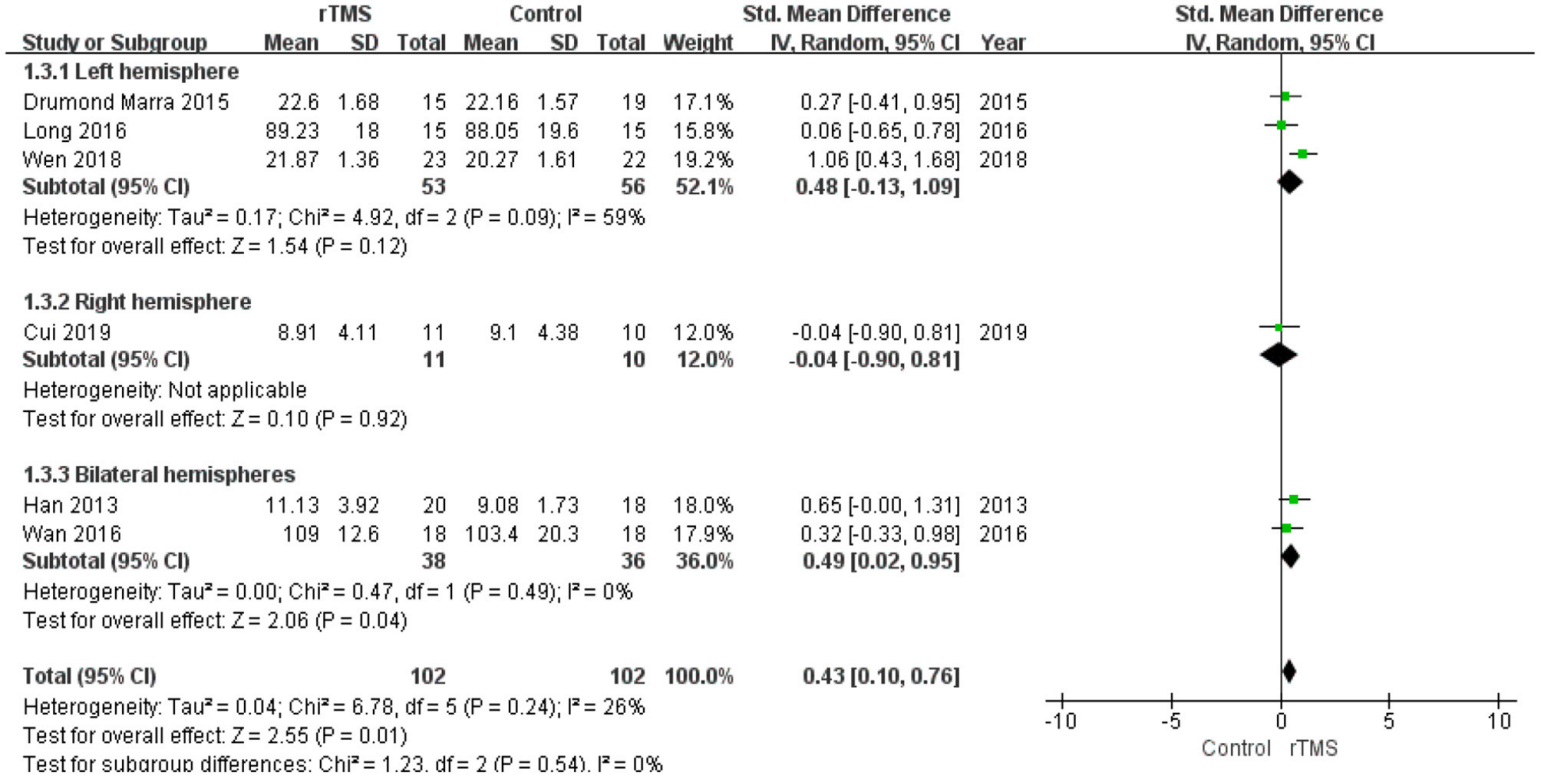
Meta-analysis forest plot of subgroup analysis showing memory of repetitive transcranial magnetic stimulation group vs. control group in the treatment of mild cognitive impairment: left hemisphere vs. right hemisphere vs. bilateral hemispheres.

A subgroup analysis was conducted according to the stimulation site (the left hemisphere, right hemisphere, and bilateral hemispheres). The subgroup analysis revealed a standardized mean difference of 0.48 (95% CI −0.13 to 1.09) for trials involving “left hemisphere” stimulation and −0.04 (95% CI −0.90 to 0.81) for trials involving “right hemisphere” stimulation. The standardized mean difference for trials involving “bilateral hemispheres” stimulation was 0.49 (95% CI 0.02 to 0.95). No significant difference in the effect size of rTMS for MCI was observed in this subgroup analysis (Figure 8).

**Figure 9:**
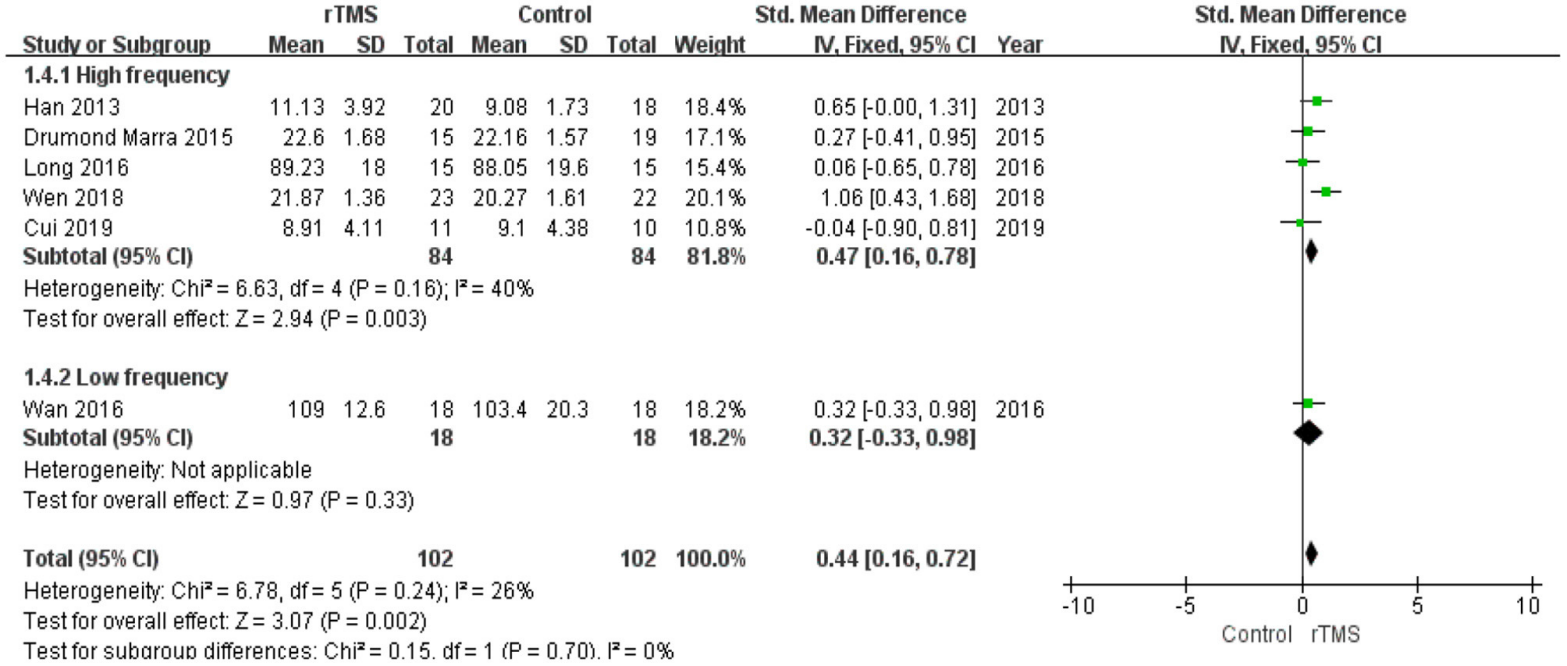
Meta-analysis forest plot of subgroup analysis showing memory of repetitive transcranial magnetic stimulation group vs. control group in the treatment of mild cognitive impairment: high frequency stimulation vs. low frequency stimulation.

The studies were divided into two groups according to the administered frequency of stimulation: a high-frequency stimulation group (≥5 Hz) and a low-frequency stimulation group (≤1 Hz). This subgroup analysis revealed no significant difference between the high-frequency subgroup (SMD 0.47, 95% CI 0.16 to 0.78, *p* = 0.003) and low-frequency subgroup (SMD 0.32, 95% CI −0.33 to 0.98, *p* = 0.33) (Figure 9).

**Figure 10:**
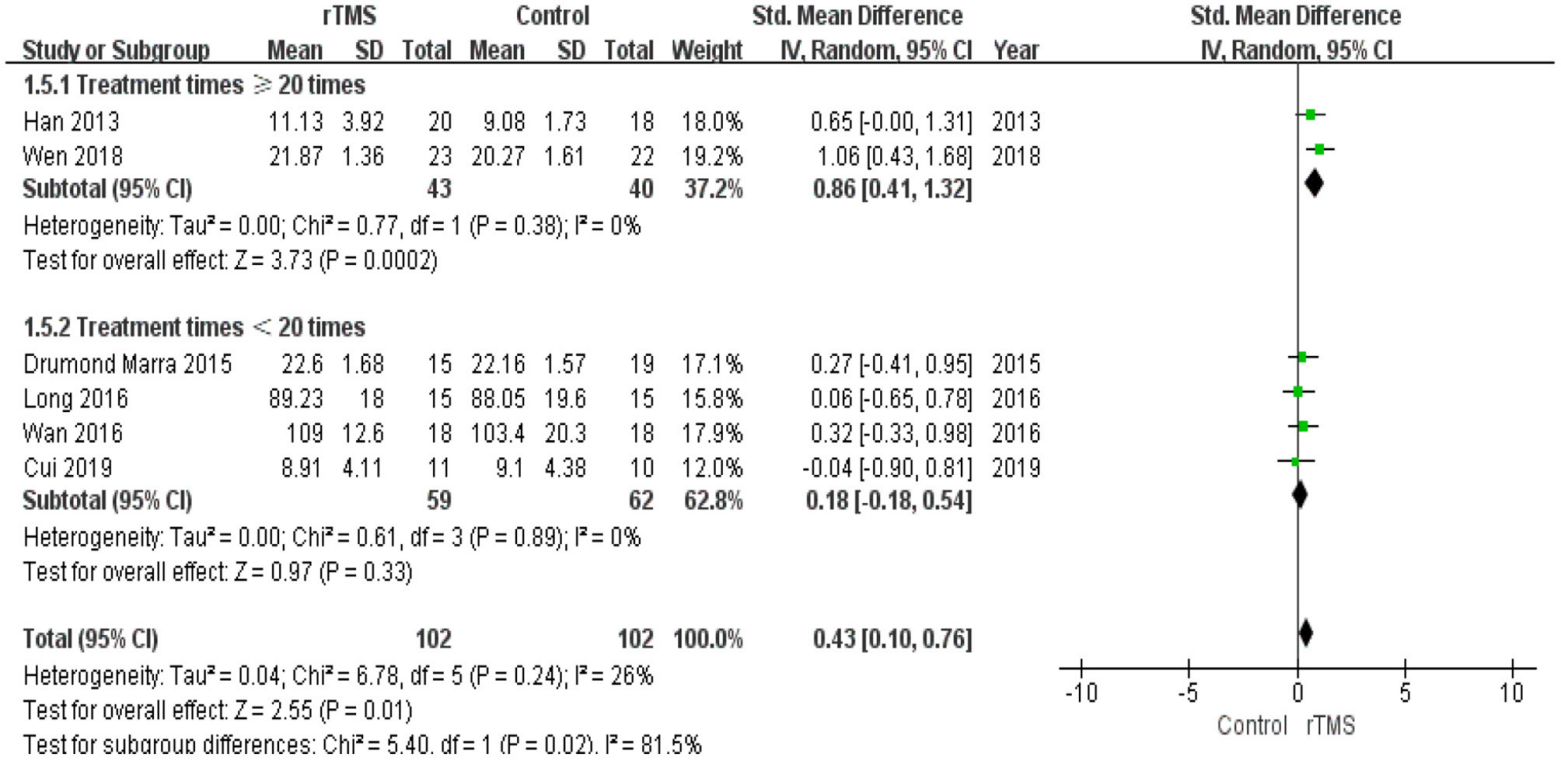
Meta-analysis forest plot of subgroup analysis showing memory of repetitive transcranial magnetic stimulation group vs. control group in the treatment of mild cognitive impairment: treatment times ≥20 times vs. treatment times < 20 times.

Subgroup analysis of the number of treatment sessions showed that participants in the high number of treatments had a significant improvement (*p* = 0.02) in memory compared with the controls (Figure 10).

#### Executive Function and Attention

The effects of rTMS on executive function and attention were measured in three studies. The heterogeneity of the included studies was low (*I*^2^ = 49%, *p* = 0.14), so a fixed-effects model was used for the meta-analysis. Meta-analysis showed no significant differences (SMD −0.19, 95% CI −0.61 to 0.22, *p* = 0.36, three studies, *n* = 93; moderate-quality evidence) between the rTMS and control groups (Supplementary Figure 1).

### Meta-Analysis of Dropout Rate

Six studies comprising a total sample size of 260 cases reported dropouts. Based on the results of the heterogeneity test (*I*^2^= 7%, *p* = 0.37), we used a fixed-effects model. The pooled results showed no differences in dropout rate between the rTMS group and the control group (RR 0.99, 95% CI 0.39 to 2.54, *p* = 0.99, six studies, *n* = 260; moderate-quality evidence) (Supplementary Figure 2).

### Meta-Analysis of Adverse Effects

None of the included studies reported serious adverse effects. Seven studies reported mild adverse effects. Discomfort was reported by 26 of the 160 patients in the rTMS group and 12 of the 157 patients in the control group. Other adverse effects reported included headache, dizziness, pain in the stimulated area, tinnitus, cervical pain, and concentration difficulties. No heterogeneity was found among the studies (*I*^2^ = 0%, *p* = 0.66). The pooled results showed that the rTMS group had a higher incidence of adverse effects than control group (RR 2.03, 95% CI 1.16 to 3.52, *p* = 0.01, seven studies, *n* = 317; moderate-quality evidence) (Supplementary Figure 3).

## Discussion

### Main Findings

This systematic review identified nine studies comprising a total of 369 participants with MCI, of which 187 were treated with active rTMS for 10 to 48 times and 182 were treated with sham rTMS, cognitive training, or drug treatment. Overall, the results of our meta-analysis support the benefits of rTMS on global cognitive function and memory in patients with MCI. To summarize all the included studies in this review, the stimulation parameters that appear more helpful in cognitive improvement in MCI patients include high frequency (5–20 Hz) rTMS applied over the left or bilateral hemisphere (especially DLPFC or PFC) for ≥ 20 treatment sessions with a stimulation intensity from 80 to 110% resting MT. Further, rTMS was found to be safe with no serious adverse effects reported.

To minimize potential heterogeneity, subgroup analyses were performed according to the stimulation site, stimulus frequency, and treatment session number. The most common stimulation target for improving cognitive function in patients with AD was the DLPFC (Cotelli et al., 2011; Liao et al., 2015). In addition to the DLPFC, the anterior temporal lobe and PFC were also targeting for rTMS, and both showed initial promising results (Zhang et al., 2014; Wan et al., 2016). It is widely known that the PFC plays a critical role in cognitive functions (Iimori et al., 2018), which are abnormally disturbed in MCI (Sanford, 2017). Further data are required to elucidate the optimal target site of rTMS for MCI. Our findings showed that high-frequency rTMS significantly enhanced global cognitive function and memory, whereas only one trial utilizing low-frequency rTMS (Wan et al., 2016) showed no significant difference in comparison with the control group (medication treatment). Consistent with previous studies, we found that high-frequency rTMS was effective on cognitive function (Nardone et al., 2014; Dong et al., 2018). However, in a single-session study (Turriziani et al., 2012), low-frequency rTMS of the right DLPFC enhanced recognition memory in eight subjects with MCI. Turriziani et al. (2012) speculated that inhibition of the right DLPFC may modulate the activity of this dysfunctional network, restoring an adaptive equilibrium in MCI. All included studies assessing changes in cognition immediately after the intervention suggested that a high number of rTMS treatments (≥20 times) produced better global cognitive function and memory effects in MCI. This meta-analysis is consistent with a previous study that suggested that a longer duration of treatment was more effective in patients with AD (Lin et al., 2019; Wang et al., 2020b). However, these results should be interpreted with caution due to the relatively small number of trials, particularly for low-frequency rTMS. Additionally, the GRADE level of evidences for the most of cognitive outcome measures were rated as “moderate”; thus, higher quality studies are required to investigate the use of rTMS for MCI.

Stimulus intensity is determined on the basis of resting MT, which mostly ranges from 80 to 110% resting MT. Previous studies revealed that the distance between the scalp and motor cortex increases linearly with MT (Herbsman et al., 2009), and the scalp-cortex distance alone may account for 60% of the variance in measurements of MT (Sabesan et al., 2015). Due to age-related brain atrophy, the scalp-cortex distance in older adults increases, thus the stimulus intensity should be adjusted considering the rate of cortical atrophy. Jorge et al. (2008) noted that the delivery of a high number of pulses was more effective than those who received smaller number of pulses a day in the elderly patients with depression. The appropriate stimulus intensity and number of pulses delivered should be systematically explored among the elderly in future studies (Iriarte and George, 2018). In addition, a wide variety of cognitive measurement tools across studies also contributed to the variation in research results. Because of multifactorial nature of neuropsychological tests, and not easily classified into single domains, thus distinct cognition classification systems have been used to facilitate interpretation. Both executive function and attention are complex and multi-dimensional cognitive constructs, and attention may be considered a specific example of executive function (Yogev-Seligmann et al., 2008; Schmitt et al., 2019). We classified the neuropsychological tests into three cognitive domains, and combined executive function with attention cognitive domains to provide a general framework in this meta-analysis (Karssemeijer et al., 2017).

rTMS is a promising, non-invasive treatment for the improvement of cognitive function in elderly patients with cognitive impairment. Recently, some studies (Ren et al., 2017; Cui et al., 2019) have also combined TMS with neuroimaging and genetic approaches to further understand the potential mechanisms underlying the rTMS effects on cognition. The default mode network (DMN), serum lipid levels (such as cholesterol and triglyceride levels) (Weng et al., 2018; Wang et al., 2020a; Yang et al., 2020), and oxidative stress (such as superoxide dismutase) (Zhu et al., 2019) have been reported to play critical roles in modulating cognitive function in age-related neurodegenerative diseases. Cui et al. (2019) showed that rTMS-induced hypoconnectivity within DMN was associated with clinical cognitive improvements in patients with amnestic MCI. High-frequency rTMS reportedly decreased serum lipid levels (including the total cholesterol and triglyceride) in the healthy older adults (Ren et al., 2017). A review presents that the therapeutic effect of TMS could be mediated at least partly by its effects on antioxidant enzymes (Medina-Fernández et al., 2018). However, MCI is still a heterogeneous clinical construct, typically divided into amnestic or non-amnestic types. Conventional classification system had a limitation, which combined patients with very different cognitive profiles (Edmonds et al., 2016). Therefore, the exploration of cluster analysis may hold great potential in finding robustly replicable subtypes (Qian and Huang, 2019; Freitas, 2020). Future studies combining TMS parameters with successful MCI subtyping and markers in imaging, biochemical, and genetic are useful to achieve more effective disease-modifying therapies (Oulas et al., 2019; Xie et al., 2019).

With respect to treatment acceptance, the present results suggested that rTMS treatment was well-tolerated, and there was no significant difference in dropout rate between the rTMS and control groups. The reason for the loss to follow-up was primarily unrelated to rTMS treatment, and none of the included trials reported serious adverse effects. On the other hand, the results of this study also suggest that rTMS treatment has better safety compared to control group. Although patients in the rTMS treatment group were found to be more prone to adverse effects than those in the control group, the adverse effects associated with rTMS were rare and mild. The most common were transient headache and dizziness.

### Limitations

A few limitations should be considered when interpreting the findings of the current study. First, the sample sizes of the included studies were small, ranging from 21 to 80 participants, which may limit the statistical power to detect the effects of rTMS on cognitive function in MCI patients. Second, there was considerable heterogeneity in the included studies with respect to the stimulation parameters (frequency, intensity, and pulses). Therefore, the optimal rTMS parameters are unclear. Finally, most of the included trials were conducted in China, only one trial was from Brazil, which may have resulted in a certain degree of language selection bias. In addition, there was another possible selection bias, since only five studies clearly described random sequence generation and one study described allocation concealment in detail (Yuan et al., 2020).

### Implications

In conclusion, the present meta-analysis study may suggest a favorable effect of rTMS on cognitive function in patients with MCI. However, there are many parameters that may affect the treatment, such as the intensity of the stimulus, frequency of the stimulus train, the site for stimulation, or even the course of treatment. Further studies should focus on the mechanism and the optimal parameter setting of rTMS, which will be of great importance for the development of this new intervention in clinical practice.

## Data Availability Statement

The original contributions presented in the study are included in the article/Supplementary Materials, further inquiries can be directed to the corresponding author/s.

## Author Contributions

LJ and CZ were responsible for the literature screening and data extraction. XC, NG, and YZ were responsible for risk of bias assessment. LJ and HC were responsible for statistical analysis and writing up the article. JW, ZY, and CL were responsible for planning and guidance on this paper. All authors contributed to the article and approved the submitted version.

## Conflict of Interest

The authors declare that the research was conducted in the absence of any commercial or financial relationships that could be construed as a potential conflict of interest.
