# Supplementary material for: Repetitive Transcranial Magnetic Stimulation for Improving Cognitive Function in Patients With Mild Cognitive Impairment: A Systematic Review
Source: Front Aging Neurosci. 2021 Jan 14;12:593000. doi: 10.3389/fnagi.2020.593000 (PMC7842279; doi:10.3389/fnagi.2020.593000)
Supplement: Supplementary file 1 [file Data_Sheet_1.docx]

**Supplementary Materials**

**
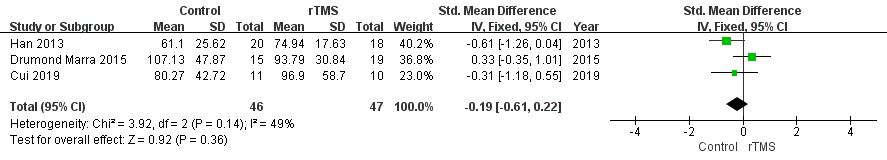
**

**Supplementary Figure 1.** Forest plot of the comparison between the repetitive transcranial magnetic stimulation and control groups with respect to executive function and attention.

**
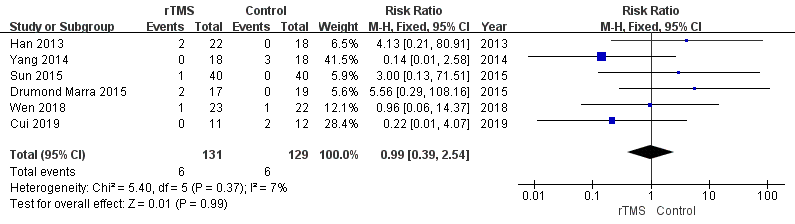
**

**Supplementary Figure 2.** Forest plot showing the dropout rate of the repetitive transcranial magnetic stimulation and control groups in the treatment of mild cognitive impairment.

**
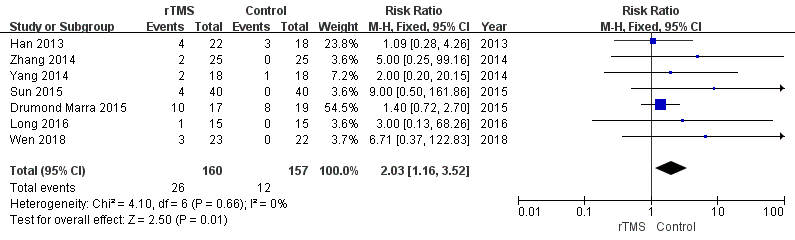
**

**Supplementary Figure 3.** Forest plot showing adverse effects in the repetitive transcranial magnetic stimulation and control groups in the treatment of mild cognitive impairment.
